# NF2/Merlin in hereditary neurofibromatosis 2 versus cancer: biologic mechanisms and clinical associations

**DOI:** 10.18632/oncotarget.1557

**Published:** 2013-12-17

**Authors:** Rebecca Dunbar Schroeder, Laura S. Angelo, Razelle Kurzrock

**Affiliations:** ^1^ Department of Investigational Cancer Therapeutics (Phase I Program), The University of Texas MD Anderson Cancer Center, Houston, TX,; ^2^ Program in Experimental Therapeutics, The University of Texas Graduate School of Biomedical Sciences, Houston, TX

**Keywords:** Neurofibromatosis 2, schwannoma, merlin, mutation

## Abstract

Inactivating germline mutations in the tumor suppressor gene NF2 cause the hereditary syndrome neurofibromatosis 2, which is characterized by the development of neoplasms of the nervous system, most notably bilateral vestibular schwannoma. Somatic NF2 mutations have also been reported in a variety of cancers, but interestingly these mutations do not cause the same tumors that are common in hereditary neurofibromatosis 2, even though the same gene is involved and there is overlap in the site of mutations. This review highlights cancers in which somatic NF2 mutations have been found, the cell signaling pathways involving NF2/merlin, current clinical trials treating neurofibromatosis 2 patients, and preclinical findings that promise to lead to new targeted therapies for both cancers harboring NF2 mutations and neurofibromatosis 2 patients.

## INTRODUCTION

**Figure 1:**
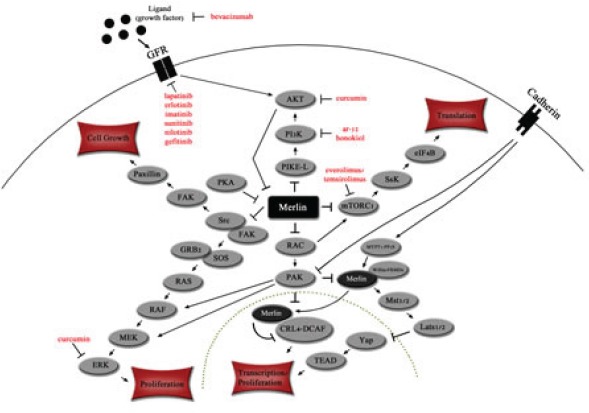
Merlin affects key signaling pathways in the cytoplasm and nucleus Merlin inhibits PI3K signaling cascade at PIKE-L[19, 30] and mTORC1[32, 33]. The RAS and Src pathways are inhibited by merlin via the Src/FAK complex[34, 36]. Transcription is inhibited by merlin via Hippo kinase cascade[81, 82] and CRL4-DCAF[83]. Merlin can also down-regulates cell surface growth factor receptors. Dotted line indicates nuclear membrane. Targeted therapies are indicated in red.

Neurofibromatosis type 2 (NF2) is a tumor suppressor gene on chromosome 22q12 that encodes a protein product named “merlin” (or schwannomin) affecting multiple cell signaling pathways (Figure 1)[1, 2]. Constitutional mutations in the NF2 gene cause an autosomal-dominant disorder (neurofibromatosis type 2) affecting about 1 in 33,000 people, and characterized by the development of tumors primarily affecting the nervous system[3]. Approximately half of the cases are due to de novo mutations not inherited from a family member [4]. NF2 mutations in neurofibromatosis 2 can either be germline (70%) or somatic mosaic (30%), the latter referring to mutations present in only a subset of cells[5, 6].

Neurofibromatosis 2 follows the Knudson 2-hit hypothesis, with constitutional (germline) mutations occurring initially (first hit) and additional somatic mutations (second hit) being required for loss of heterozygosity and tumor suppressor inactivation followed by tumor formation[7-9]. The majority of tumors associated with neurofibromatosis type 2 are schwannomas, meningiomas, and ependymomas[7]. Other than these specific tumor types, there is no published evidence to support an increased incidence of cancer in individuals with neurofibromatosis 2[7]. In general, in neurofibromatosis 2, constitutional nonsense or frameshift NF2 mutations are associated with more severe disease, while missense mutations, large deletions, or somatic mosaicism results in milder disease (fewer tumors and older age of onset)

**Figure 2:**
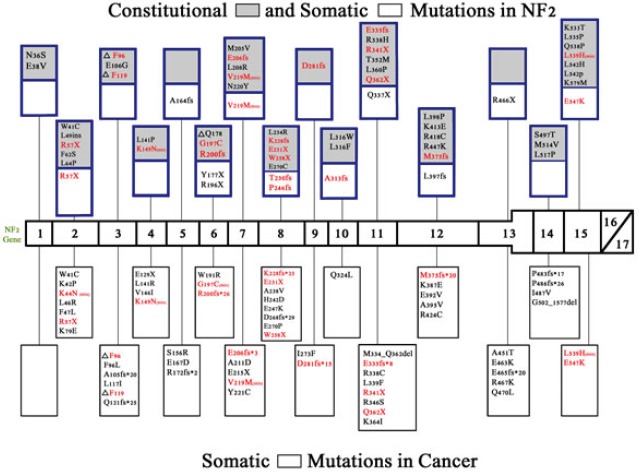
Constitutional and somatic NF2 mutations in neurofibromatosis 2 and somatic NF2 mutations in cancer NF2 mutations have been found in neurofibromatosis 2 and also in numerous cancers[15]. Shown are the non-truncating constitutional and somatic mutations found in neurofibromatosis 2 [blue boxes] and non-truncating (missense) and truncating (nonsense or frameshift) mutations found in human cancer [black boxes][84]. Gray boxes indicate constitutional mutations while white boxes indicate somatic mutations. Because there are numerous truncating lesions found in neurofibromatosis 2[69] and in non-hereditary meningiomas, ependymomas, and schwannomas,[69, 85] these could not be depicted in figure 2. Truncating lesions that are found in both neurofibromatosis 2 and cancer are shown in red. Missense mutations that are found in both neurofibromatosis 2 and cancer are also shown in red followed by [mis]. Termination sites of translation for frameshift mutations is indicated with an (*) and for nonsense mutations with an (X).

NF2 somatic mutations have also been found in multiple cancer types, including but not limited to mesothelioma, anaplastic thyroid cancer, breast cancers, endometrial and liver cancers, in patients not having constitutional NF2 mutations (Table 1)[10]. Missense mutations are more frequent in cancer than in neurofibromatosis 2; they occur in only a small subset of patients with latter condition. This may in part explain the lack of predisposition to developing cancer in patients with neurofibromatosis 2, though the explanation is not complete, since there remains considerable overlap between neurofibromatosis 2 and cancer-related NF2 aberrations (Figure 2)[11, 12].

**Table 1:** Cancers associated with NF2 somatic mutations

Cancer Type	% Containing NF2 Somatic Mutations	Source
Acute myelogenous leukemia (AML)	2%	(1/45)	Yoo et al, Pathology 2012 [9] COSMIC
Aerodigestive tract (Squamous cell carcinoma)	4%	(1/23)	COSMIC
Bladder	3%	(1/14)	Iyer G et al., Science 2012 [66]
Bone (sarcoma)	6%	(2/34)	COSMIC
Breast Cancer	1-2%	(3/387) (1/42)	COSMIC ICGC Data Portal (http://dcc.icgc.org) Bianchi et al, Nature Genetics 1994[13] Sjöblom T et al Science 2006 [63] Ikediobi et al, Mol Cancer Ther 2006[64]
Ependymoma*	4%	(18/433)	COSMIC
Colorectal Carcinoma	5%	(2/44)	Arakawa H et al, HMG 1994 [87]
Endometrium (mixed adenosquamous carcinoma)	10%	(1/10)	COSMIC
Glioma	27%	(37/135)	Lau YK et al, Cancer Res 2008[25]
Hepatocellular Carcinoma (HCC)	2%	(1/45)	Yoo et al, Pathology 2012 [9] COSMIC
Intestine (large)	2%	(8/335)	COSMIC
Liver Cancer	23%	(17/75)	ICGC Data Portal (http://dcc.icgc.org)
Lung (Adenocarcinoma)	1%	(1/163)	ICGC Data Portal (http://dcc.icgc.org)
Lung (Mixed)	1%	(3/586)	COSMIC
Lung (Squamous Cell Carcinoma)	2%	(1/45)	Yoo et al, Pathology 2012 [9] COSMIC
Melanoma	5%	(6/126)	COSMIC Bianchi et al, Nature Genetics 1994 [13]
Meningioma *	31%	(363/1164)	COSMIC
Mesothelioma	30-50%	(8/27)	COSMIC Carbone and Yang, CCR 2012 [88] Sekido Y, et al, Cancer Res 1995 [89] Bianchi AB, et al. Proc Natl Acad Sci 1995 [90]
Ovary (serous carcinoma)	1%	(2/149)	COSMIC
Pituitary	100%	(1/1)	COSMIC Szijan et al, Neuromolecular Med 2003 [91]
Renal cell carcinoma	1-2%	(5/412) (7/428)	Staller P, Future Oncology 2010 [92] COSMIC
Schwannoma *	42%	(271/647)	COSMIC
Stomach	3%	(2/66)	COSMIC
Thyroid (anaplastic carcinoma)	18%	(2/11)	COSMIC
Urinary tract	11%	(2/18)	COSMIC

* Sporacic tumors, patients do not have neurofibromatosis 2 COSMIC (http://www.sanger.ac.uk/cosmic)

### Clinical Manifestations of Neurofibromatosis 2

The most common tumor associated with neurofibromatosis 2 is bilateral vestibular schwannoma (acoustic neuromas)[12, 13]. Initial symptoms of neurofibromatosis 2 include hearing loss, facial nerve impairment, visual disruptions, and skin tumors; with the majority of patients presenting before the age of 20[12]. Many patients with multiple tumor sites live only into their early thirties, and overall survival rate for patients 20 years after diagnosis is approximately 40%[12, 13]. Treatment plans for patients with NF2 revolve around monitoring, surgery, and radiation therapy[12]. Due to recent advancements in our understanding of the signaling pathways affected by merlin, several targeted therapies are now being tested[14]. However, there remains a paucity of clinical trials, and a pressing need to accelerate the pace of testing in the clinical setting.

### Merlin

The NF2 gene contains 17 exons and its product— merlin—is a tumor suppressor that controls protean cell signaling pathways implicated in cell growth, proliferation, and morphology[15, 16]. Merlin acts as a membrane-cytoskeleton scaffolding (Ezrin–Radixin– Moesin (ERM)) protein that localizes underneath the plasma membrane at cell–cell junctions and other actinrich sites[17, 18]. It links the cytoskeleton to the cell membrane. Two isoforms of merlin have been described that differ by the presence (type 2 merlin) or absence (type 1 merlin; includes exons 1-15 and 17) of exon 16 sequences inserted into the extreme carboxyl terminus of the protein.

Myosin phosphatase MYPT1–PP1δ directly activates merlin by dephosphorylating it. Inactivation (phosphorylation) of merlin contributes to malignant conversion in multiple cell types, as does loss of merlin expression because of mutation [19]. Importantly, phosphorylation not only causes the conformation of merlin to change to the open/inactive state, but also targets merlin for polyubiquitination and degradation by the proteasome [19-21]. The decreased ability of mutant merlin to effectively act as a tumor suppressor is at least in part a direct result of the decreased half-life of mutant compared to wild-type merlin[22, 23].

### Functions of NF2/merlin

Several different signaling pathways crucial to cell proliferation are inhibited by merlin: PIKE-L/PI3K, mTORC1, Src/Fak, Mst1/2, Ras/Rac/PAK, ERK1/2, AKT and CRL4-DCAF (Figure 1)[1, 24, 25]. Merlin also acts as an unconventional cell cycle regulator, and it links receptors at the plasma membrane to their cytoplasmic kinases to facilitate contact inhibition [26, 27].

#### PI3K/PIKE-L

The phosphatidy linositol 3-kinase (PI3K) pathway is activated in multiple human cancers, and contributes to increased cellular proliferation and metabolism, and decreased apoptosis when activated[28, 29]. Merlin suppresses the PI3K pathway by binding with the PI3K-enhancer-isoform-L (PIKE-L), hence preventing PIKE-L from binding PI3K[30, 31].

#### mTORC1

Inhibition of mammalian-target-of-rapamycin-complex-1 (mTORC1) due to merlin activation leads to the inhibition of mRNA translation, which causes an increase in apoptosis limiting cell survival and blocking tumor initiation[32]. mTORC1 inhibition is accomplished through multiple pathways including AKT/ERK inhibition and integrin specifc adhesion via p21-activated kinase (PAK)[32, 33]. mTORC1 has also been identified as an important pathway in meningioma and schwannoma cell growth[33].

#### ErbB2/Src/FAK/Paxillin

Merlin binds the receptor ErbB2 and also to Src, inhibiting Src from binding to and being phosphorylated by ErbB2 in a competitive manner [34]. As a result, Src cannot activate focal adhesion kinase (FAK) and paxillin, which inhibits cellular proliferation and growth[34]. Interestingly, binding of merlin to ErbB2 is initiated by paxillin binding to merlin on exon 2 at residues 50-70, causing merlin to move to the plasma membrane where it binds ErbB2[35]. Merlin also inhibits FAK from binding to Src and PI3K [36].

#### ERK1/2, AKT, and PDGFR

Schwannoma cell lines lacking the tumor suppressor activity of merlin have high basal levels of phosphorylated extracellular signal-regulated kinase (ERK1/2) and AKT, which are activated by the Src/FAK/Ras and PI3K/Raf signaling cascades and platelet-derived growth factor receptor beta (PDGFRβ) (Figure 1)[37]. Merlin inhibits the activity of ERK and MAPK via the upstream effector Raf-1[38, 39]. In normal cells, merlin promotes PDGFRβ degradation as well, thereby inhibiting cellular proliferation [37, 40]. In a positive feedback loop, activated AKT also binds and phosphorylates merlin on residues Thr230 and Ser315, which causes merlin to be ubiquitinated and targeted for degradation[19, 20, 41].

#### Rac/PAK

Due to merlin's sequence homology with ERM proteins, merlin binds the same proteins, but unlike ERM proteins merlin acts in an inhibitory manner by suppressing cell growth and proliferation [42, 43]. Merlin may suppress the oncogenic signaling of the small GTPases Ras and Rac by interfering with guanine nucleotide-exchange factor (GEF) activity, which is required for Ras and Rac activation[43, 44]. Merlin inhibits signaling of the Ras pathway downstream by inhibiting Rac activation, which results in the inhibition of phosphorylation/activation of the serine/threonine kinase PAK [43]. When PAK is not phosphorylated it is unable to phosphorylate RAF and MEK, which are both necessary to activate the Ras signaling pathway[43, 45].

### NF2 mutations in cancer

Data collected from COSMIC and ICGC databases and published studies show that diverse cancers harbor somatic NF2 mutations (Table 1).

#### Thoracic tumors (mesothelioma and lung cancer)

The most common cancer linked to NF2 aberrations is mesothelioma, with approximately 30-50% of tumors having mutations in the NF2 coding regio[22, 46-48]. The NF2 mutations found in neurofbromatosis 2 differ from the mutations found in mesotheliomas in that mutations in hereditary NF2 are not usually missense mutations. Only ~5% of neurofibromatosis 2 patients have constitutional missense mutations and these types of mutations are typically associated with a milder version of NF2 [49, 50]. This dichotomy may partially explain why patients with neurofibromatosis 2 do not develop mesothelioma[51]. A variety of lung cancers have also been found to harbor NF2 mutations, but at a much lower rate (1-2%)[11].

#### Sporadic schwannomas, meningiomas, and ependymomas

Sporadic schwannomas, meningiomas, and ependymomas fund in patients who do not suffer from constitutional neurofibromatosis 2 have somatic NF2 mutations at rates of 42%, 27%, and 4%, respectively[46]. These are also the most prevalent tumor types found in individuals with constitutional NF2 mutations[7]. Mutations found in these sporadic tumors tend to include more frameshift mutations, while those found in neurofibromatosis 2 are often nonsense mutations[52, 53].

#### Thyroid cancer

NF2 mutations have also been discerned in 18% (two of 11) anaplastic thyroid cancers. These are uncommon (accounting for only 2% of all thyroid cancers) but very aggressive cancers, with a median survival of only a few months[54]. Of interest, other pathways (PIK3/AKT/MTOR, RAS/RAK and ERK) activated via mutation in this cancer type are negatively regulated by active merlin [54-59].

#### Breast cancer

One to two percent of breast cancers have NF2 mutations [15, 60-62]. A decrease in merlin expression correlates with increase in tumor grade [47]. When merlin expression is reestablished in breast xenograft models, tumorigenesis is reduced [47]. Since NF2 mutations activate mTor, we treated a patient with NF2-mutant metaplastic breast cancer (a highly refractory form of breast cancer) with a temsirolimus-based regimen, which resulted in a complete remission [63].

#### Glioblastoma multiforme

Decreased expression of merlin RNA and protein levels have been observed in several human glioblastoma multiforme tumors; 27% of grade 4 gliomas have loss of merlin expression [46]. Merlin decreases proliferation and invasiveness while increasing apoptosis-induced cell death when its tumor suppressor capabilities are reestablished in the corresponding glioma cell lines [46].

#### Endometrial cancer

Ten percent of endometrial carcinomas harbor NF2 mutations. This observation is of interest since NF2 mutations can activate mTor, the downstream effector of PIK3CA, and a high percentage of this cancer type (>80%) have PI3K pathway aberrations [30, 64].

#### Hepatocellular tumors

Twenty-three percent of liver cancers have mutations in NF2. Mice with heterozygous NF2 mutations develop hepatocellular carcinoma and cholangiocarcinoma [65, 66].

### Comparing NF2 aberrations in hereditary neurofibromatosis and in cancer

Overlap in truncating and non-truncating mutations found in cancers and hereditary neurofibromatosis 2 are shown in Figure 2 (red lettering). There have not been any mutations found in exons 16 and 17, which are the two exons affected by alternative splicing, and no known proteins have been found to bind to exon 16[33, 67]. Tumor suppressor activity can be attenuated by truncating mutations in any of the other exons 1-15[68]. In neurofibromatosis 2, truncating mutations (frameshift and nonsense) are associated with an increase in disease severity when compared with missense mutations[7]. Nonsense mutations are more frequent in hereditary neurofibromatosis 2 than in sporadic schwannomas, ependymomas, and meningiomas where frameshift mutations are more common [53, 69]. Missense mutations are more frequent in cancer, but occur in only about 5% of hereditary neurofibromatosis 2.

There are several binding sites in the merlin protein that are crucial for merlin's function, being critical to interactions with other proteins or for correct merlin folding and activation. Merlin must associate with itself in order to change conformation and become active. This is achieved first by the folding of the N-terminal domain, which involves the interaction of amino acids 8-121 with amino acids 200-320[67, 70]. After the conformational change of the N-terminus is complete, merlin can transition to the active, closed conformation by the association of the N-terminal amino acids 302-308 with the C-terminal amino acids 580-595[67, 70]. When examining Figure 2, it is remarkable that no mutations are found in the region where the C-terminus and N-terminus interact, but multiple mutations are found in the regions where the N-terminus interacts with itself.

The phosphatase (MYPT1-PP1δ) responsible for the dephosphorylation and activation of merlin (at residue S518) interacts with merlin via its MYPT1 subunit at residues 312-341 of merlin (exons 10 and 11)[67]. As shown in figure 2, this region has multiple missense mutations in both cancer and NF2.

P21-activating kinases (PAK) are regulated by merlin and also regulate the activity of merlin by phosphorylating S518 and causing merlin to change to the open, inactive state[67]. PAKs interact with merlin at the N-terminus between amino acids 1-313, and there are many missense mutations in both cancer and neurofibromatosis 2 found within this region (Figure 2) [67].

PIKE-L binds to merlin in the region containing amino acids 1-332, whereas mutant merlin harboring the L64P missense mutation does not bind PIKE-L[30, 67]. Importantly, merlin cannot suppress tumorigenesis, including cellular proliferation, via inhibition of the PI3K pathway without binding to PIKE-L[30, 67]. Merlin's interaction with PIKE-L could also regulate downstream effectors of PI3K such as AKT and mTOR[67]. The L64P mutation has not been found in any cancers but is reported in neurofibromatosis 2.

### Clinical Trials

Novel targeted therapies are now being used in a small number of cases to treat hereditary neurofibromatosis 2 and cancers harboring NF2 mutations (Table 2). The vast majority of these trials are ongoing and results are not yet published. A search of clinicaltrials.gov was undertaken in order to find clinical trials treating NF2 patients.

**Table 2:** Ongoing Clinical Trials for Neurofibromatosis 2 Patients

Drug Name	Mechanism	Phase of Study	Trial Reference Number
Lapatinib	Dual tyrosine kinase inhibitor (Her2/EGFR)	Phase 0	NCT00863122
PD-0332991	CDK inhibitor	Phase 1	NCT01602887
Bevacizumab	Anti-VEGF monoclonal antibody	Phase 2	NCT01207687, NCT01125046
Everolimus (RAD-001)	mTOR inhibitor	Phase 2	NCT01490476, NCT01345136, NCT01419639
Lapatinib	ErbB2/EGFR inhibitor	Phase 2	NCT00973739
Nilotinib	Receptor tyrosine kinase inhibitor (Bcr-Abl, PDGFR, c-KIT)	Phase 2	NCT01201538
PTC299	Decrease production of VEGF	Phase 2	NCT00911248
Sunitinib	Receptor tyrosine kinase inhibitor (PDGFR, VEGFR, c-KIT)	Phase 2	NCT00561665, NCT00589784

#### Receptor tyrosine kinase (RTK) Inhibitors

Lapatinib, which targets EGFR and ErbB-2, is being examined in both a phase 0 study treating vestibular schwannomas (NCT00863122) and a phase II (NCT00973739) trial for its effectiveness in all NF2 related tumor types. Clinical analysis has shown that vestibular schwannomas overexpress ErbB2/3 and that EGFR and its ligand are up-regulated in the majority of NF2-related vestibular schwannomas[71, 72].

Sporadic and neurofibromatosis 2-related vestibular schwannomas overexpress c-kit and PDGFR, which are targets of the receptor tyrosine kinase (RTK) inhibitor imatinib. In vitro studies using the vestibular schwannoma cell line HEI-193 show a decrease in proliferation and an increase in apoptosis in response to treatment with imatinib. Currently sunitinib and nilotinib, are being tested in patients with neurofibromatosis 2[13]. Sunitinib is an RTK inhibitor that targets multiple receptors such as PDGFR, VEGFR, and c-KIT. Two phase II clinical trials are currently evaluating the effectiveness of sunitinib in unresectable meningiomas (NCT00561665) and (NCT00589784). Nilotinib is a RTK inhibitor that targets Bcr-Abl, PDGFR, and c-Kit, and is also being used in a phase II clinical trial (NCT01201538) focusing on progressing vestibular schwannomas.

#### Vascular Endothelial Growth Factor (VEGF) Inhibitors

Bevacizumab is an anti-VEGF monoclonal antibody that inhibits angiogenesis, slowing tumor growth and formation. Schwannomas produce high levels of VEGF. In a study of ten NF2 patients treated with bevacizumab, nine had tumor shrinkage, and seven experienced hearing improvement. Two phase II clinical trials (NCT01207687, NCT01125046) are assessing the effectiveness of bevacizumab in treating neurofibromatosis 2 patients with symptomatic vestibular schwannomas and recurrent or progressive meningiomas. Another drug PTC299 targets VEGF by inhibiting its synthesis upstream and interfering with post-transcriptional processing [73]. PTC299 has been studied in neurofibromatosis 2 patients via a phase II clinical trial [73].

#### mTOR Inhibitors

Everolimus (RAD-001) is an mTOR inhibitor. Merlin is a negative regulator of mTOR. Therefore, in patients with deactivating NF2 mutations, one could hypothesize that an mTOR inhibitor would restore merlin's inhibition of mTOR and arrest tumor formation [32]. There are three phase II clinical trials using everolimus to treat NF2-related tumors: NCT01490476, NCT01345136 and NCT01419639. A fourth phase II clinical trial (NCT01024946) is available for treatment of malignant pleural mesotheliomas using NF2/merlin loss as a biomarker to predict everolimus sensitivity. The mTOR inhibitor temsirolimus was tried in combination with the anti-VEGF antibody bezacizumab in a clinical case series focusing on patients with neurofibromatosis 2; of the two patients on this regimen, one achieved a 33% reduction in tumor size[74]. Of interest, one patient with metaplastic breast cancer who harbored an NF2 mutation achieved a complete remission on a temsirolimus-containing regimen [63].

#### RAS and CDK inhibitors

Merlin blocks RAS activation. When merlin activity is lost, RAS can then move into the active state and promote cell growth and proliferation [13]. S-trans, trans-farnesyl-thiosalicylic-acid (FTS) is a RAS inhibitor that was administered to two patients with hereditary neurofibromatosis 2, one of whom achieved stable disease for over 4.5 years[74, 75].

### Preclinical Studies

Treatment of human schwannoma cells in vitro with curcumin (diferuloylmethane), caused dephosphorylation of AKT and ERK1/2 and activation of the merlin phosphatase MYPT1-pp1δ, which is responsible for the dephosphorylation of S518 [76]. Interestingly, hsp70 was up-regulated following curcumin treatment, which could serve as a resistance mechanism. Hence, a heat shock protein inhibitor (KNK437) was used in combination with curcumin to block this potential resistance mechanism [76]. Merlin loss also causes constitutive activation of receptor tyrosine kinases (EGFR, ErbB2, and ErbB3), which can be targeted by EGFR and ErbB2 inhibitors such as erlotinib or lapatinib[13]. Finally, sorafnib, a PDGFR and c-RAF inhibitor has been shown to decrease proliferation in human schwannoma cell line [37].

## CONCLUSIONS

NF2 is a complex gene with somatic mutations being associated with various cancers (e.g. 30 to 50 percent of mesotheliomas harbor NF2 mutations (Table 1)). Germline mutations cause the autosomal dominant disorder neurofibromatosis 2. Although the somatic mutations sometimes overlap with those in hereditary NF2 (Figure 2), there are no published papers documenting an increased risk of cancer in neurofibromatosis patients; individuals with hereditary NF2 do develop schwannomas, ependymomas, and meningiomas. Though there is overlap in the types of NF2 mutations/aberrations between NF2-related conditions (Figure 2), nonsense mutations are more frequent in neurofibromatosis 2, frameshift mutations in sporadic schwannomas, meningiomas, and ependymomas, and missense mutations in cancer[69].

Merlin inhibits PIK3CA and mTOR function along with several other pathways including Src/Fak, Mst1/2 (hippo), Ras/Rac/PAK, ERK1/2, AKT and CRL4-DCAF (Figure 1). A small series of mostly anecdotal reports describe activity for anti-angiogenesis agents (bevacizumab), a RAS inhibitor, and the mTor inhibitor temsirolimus in patients with hereditary neurofibromatosis 2[74, 77]. In patients with malignancy, an increasing number of studies have established that matching targeted therapy to even a single aberration in patients whose tumors harbor multiple genomic abnormalities can at times result in remarkable responses[78-80]. Since neurofibromatosis 2, unlike cancer, is a single gene disorder, it seems conceivable that proper targeting would result in tumor regressions. In order to explore this possibility, a variety of relevant agents should be explored in the clinical setting. This is especially important because of the morbidity and mortality associated with neurofibromatosis 2, and because many agents that impact NF2-related pathways are already available. Rare conditions are an accrual challenge for larger trials. Therefore, pilot trials whose aim is tumor response may be a mechanism to initially establish activity of an agent, and more of these trials are urgently warranted.
